# Initial Processes of Atomic Layer Deposition of Al_2_O_3_ on InGaAs: Interface Formation Mechanisms and Impact on Metal-Insulator-Semiconductor Device Performance

**DOI:** 10.3390/ma5030404

**Published:** 2012-03-08

**Authors:** Wipakorn Jevasuwan, Yuji Urabe, Tatsuro Maeda, Noriyuki Miyata, Tetsuji Yasuda, Hisashi Yamada, Masahiko Hata, Noriyuki Taoka, Mitsuru Takenaka, Shinichi Takagi

**Affiliations:** 1 National Institute of Advanced Industrial Science and Technology (AIST), Tsukuba, Ibaraki 305-8562, Japan; E-Mails: yuji.urabe@aist.go.jp (Y.U.); t-maeda@aist.go.jp (T.M.); nori.miyata@aist.go.jp (N.M.); yasuda-t@aist.go.jp (T.Y.); 2 Sumitomo Chemical Co., Ltd, Tsukuba, Ibaraki 300-3294, Japan; E-Mails: yamadah7@sc.sumitomo-chem.co.jp (H.Y.); hatam1@sc.sumitomo-chem.co.jp (M.H.); 3 Department of Electrical Engineering, The University of Tokyo, Bunkyo-ku, Tokyo 113-8656, Japan; E-Mails: ntaoka@mosfet.t.u-tokyo.ac.jp (N.T.); takenaka@mosfet.t.u-tokyo.ac.jp (M.T.); takagi@ee.t.u-tokyo.ac.jp (S.T.)

**Keywords:** InGaAs, Al_2_O_3_, ALD, MISFET, trimethylaluminum

## Abstract

Interface-formation processes in atomic layer deposition (ALD) of Al_2_O_3_ on InGaAs surfaces were investigated using on-line Auger electron spectroscopy. Al_2_O_3_ ALD was carried out by repeating a cycle of Al(CH_3_)_3_ (trimethylaluminum, TMA) adsorption and oxidation by H_2_O. The first two ALD cycles increased the Al KLL signal, whereas they did not increase the O KLL signal. Al_2_O_3_ bulk-film growth started from the third cycle. These observations indicated that the Al_2_O_3_/InGaAs interface was formed by reduction of the surface oxides with TMA. In order to investigate the effect of surface-oxide reduction on metal-insulator-semiconductor (MIS) properties, capacitors and field-effect transistors (FETs) were fabricated by changing the TMA dosage during the interface formation stage. The frequency dispersion of the capacitance-voltage characteristics was reduced by employing a high TMA dosage. The high TMA dosage, however, induced fixed negative charges at the MIS interface and degraded channel mobility.

## 1. Introduction

Si complementary metal-oxide-semiconductor (CMOS) circuits are approaching the physical limit of scaling, and booster technologies for equivalent scaling have been seriously explored. III-V compound semiconductors have considerable potential as a channel material for high performance n-channel metal-insulator-semiconductor field-effect transistors (MISFETs) due to their high electron mobility. Improvement of the MIS interface is a central issue in order for III-V channels to be successfully implemented in future CMOS circuits. It is widely recognized that native oxides on III-V surfaces deteriorate the MIS interface properties [1,2], and many interface control methods have been proposed to remove or modify these oxides, such as surface passivation by wet oxide etching [3,4], sulfur passivation [5,6], plasma cleaning [7,8], nitridation [9,10], Si deposition [11,12], and atomic layer deposition (ALD) using reducing reactants [13,14]. Among these studies, the effects of Al_2_O_3_ ALD on III-V surfaces have been investigated extensively [15,16]. Al_2_O_3_ ALD using Al(CH_3_)_3_ (trimethylaluminum, TMA) as the precursor has the so-called self-cleaning effect that reduces Ga and As oxides on GaAs, InAs, and InGaAs surfaces. *In situ* monochromatic x-ray photoelectron spectroscopy (XPS) measurements after every ALD half cycle have revealed the surface reactions of Al_2_O_3_ growth on GaAs and InGaAs surfaces [15,16]. The first TMA exposure dramatically reduces the Ga^3+^ and As^3+^ oxidation states below the level of XPS detection, and after ALD of an 8-nm-thick Al_2_O_3_ layer, the As^5+^ oxidation state was detected on the top of the grown Al_2_O_3_ as opposed to the As^3+^ state. This As^5+^ state disappeared after heating at 280 °C due to evaporation of AsO_x_. The capacitance-voltage (C-V) curves of Al_2_O_3_/InGaAs capacitors showed improved interface quality because of this self-cleaning process [15]. Although these studies investigated the bonding states of the surface oxides in detail, there are only a few studies on interface control by varying the ALD conditions [17,18]. *In situ* scanning tunneling microscopy (STM) and scanning tunneling spectroscopy (STS) disclosed the reaction of TMA on the group III rich (4 × 2) reconstruction of the InGaAs surface, and a more ordered reconstruction occurred when the InGaAs surface was dosed to a saturation level with TMA and subsequently annealed at 200 °C. The shift of the Fermi level after the addition of 1 monolayer of TMA for both the n-type and p-type InGaAs surfaces indicated unpinned interface formation.

In this paper, we first report results for the on-line Auger electron spectroscopy (AES) analysis of the initial growth processes of Al_2_O_3_ ALD on InGaAs. The results show clearly that the initial growth consists of an interface-formation stage and a bulk-growth stage. The former is characterized by no oxygen uptake from the vapor phase during the ALD cycles. This result prompted us to change the ALD conditions of the interface-formation stage independently from those of the bulk-growth stage. In particular, we focused on the effects of a high TMA dosage at the interface-formation stage, because the TMA dosage is thought to affect the extent of the surface-oxide reduction and to thereby change the MIS properties. The second half of this paper reports the electrical properties of MIS capacitors and MISFETs prepared by changing the TMA dosage in the first few cycles of Al_2_O_3_ ALD.

## 2. Experimental Section

The experiments were carried out using an ALD system that is high-vacuum compatible and equipped with an analysis chamber for AES [19]. The In_0.53_Ga_0.47_As epitaxial wafers were prepared by metal-organic chemical vapor deposition (MOCVD) on InP wafers. MIS capacitors and MISFETs were fabricated on n-type Si-doped and p-type Zn-doped InGaAs epitaxial layers, respectively. The use of n-InGaAs for the capacitors allows us to probe the interface properties in the upper half of the bandgap. n-InGaAs wafers were also used for the AES experiments. The thickness and doping concentration of the epitaxial layer were 0.5 µm and ~3 × 10^16^ cm^−3^, respectively, for both the p- and n-type InGaAs. Prior to ALD, the InGaAs surfaces were treated in an NH_4_OH solution and rinsed in deionized H_2_O for 1 min. The Al_2_O_3_ films were then deposited using TMA and H_2_O as precursors at 250 °C. The TMA dosage for our standard ALD conditions was ~0.005 Pa·s.

The amount of aluminum and oxygen on the InGaAs surface was monitored by on-line AES. The AES measurements were carried out after each of the following process steps: NH_4_OH etching, heating to 250 °C, and 1, 2, 3, 5, and 10 ALD cycles. For each measurement, the sample was transferred from the ALD chamber directly to the AES chamber. The electron beam energy was 5 keV. To supplement the AES results, the samples after ALD were also analyzed by *ex situ* XPS using an Al Kα x-ray source (1,486.7 eV).

In order to study the effects of a high TMA dosage on the MIS interface properties, the initial ALD cycles were carried out at a TMA dosage of ~0.5 Pa·s. The samples were prepared with this high TMA dosage for the initial cycles and with the standard dosage for the following cycles, keeping the total number of cycles at 60. The number of ALD cycles with a high TMA dosage was varied from zero to five. The growth rate of Al_2_O_3_ was 0.10 nm/cycle regardless of the TMA dosage. After completing ALD, post deposition annealing (PDA) was carried out at 400 °C for 2 min in vacuum. Evaporated Au was used as the front electrode in the capacitors.

MISFETs were fabricated on p-InGaAs using the gate-last process [5,6]. The wafer surface was first deposited with a 6-nm-thick Al_2_O_3_ protection layer. Si implantation at the source and drain regions was carried out at an acceleration energy of 30 keV and a dose of 2 × 10^14^ cm^−2^ using a photoresist mask. The dopant was activated by rapid thermal annealing in an N_2_ flow at 600 °C for 10 s. After removing the protection layer by buffered hydrofluoric (BHF) etching, the channel surface preparation and the ALD and PDA processes were carried out under the same conditions as MIS capacitor fabrication. A 30-nm-thick TaN_x_ gate electrode was then deposited on the Al_2_O_3_film by ion-beam sputtering. After opening the source and drain contact holes by selectively etching TaN_x_ and Al_2_O_3_, the Ti-Au stacks for the source, drain, and gate contacts were formed by electron beam evaporation and a lift-off process. The fabrication process was completed by post metallization annealing in an N_2_ flow at 350 °C for 90 s.

## 3. Results and Discussion

### 3.1. AES and XPS Analyses of InGaAs Surfaces

**Figure 1:**
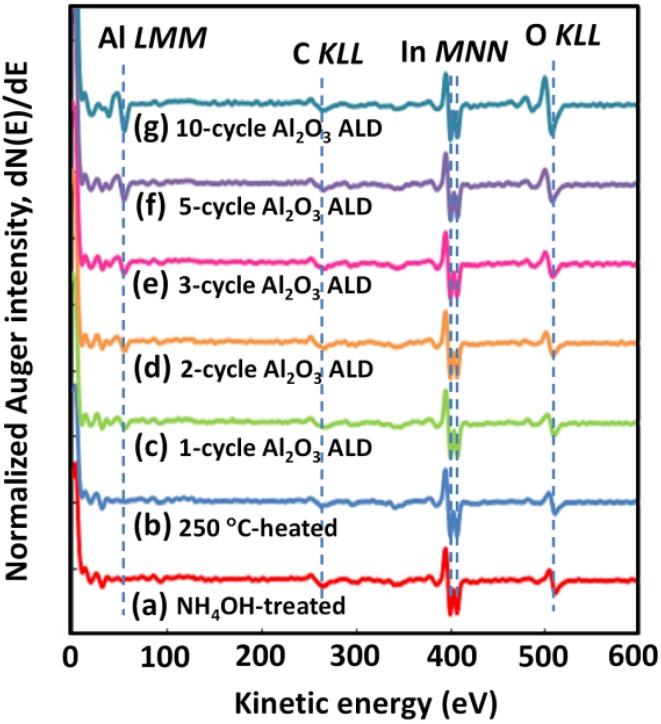
AES spectra of (**a**) NH_4_OH-treated InGaAs(100), (**b**) after heating, (**c**) 1-, (**d**) 2-, (**e**) 3-, (**f**) 5-, and (**g**) 10-cycle Al_2_O_3_ ALD at 250 °C.

Figures 1 (a–g) show a series of Auger spectra taken during the course of NH_4_OH treatment, 250 °C heating, and Al_2_O_3_ ALD with the standard TMA dosage for the first ten cycles on InGaAs(100). The spectra were normalized with respect to the In MNN intensity. NH_4_OH-treated InGaAs surfaces had a surface oxide as shown by the O KLL signal in spectrum (a). Spectrum (b), obtained after heating at 250 °C, shows that the O KLL intensity decreased slightly due to evaporation of the adsorbed H_2_O from the InGaAs surface. An increase of the O KLL intensity during Al_2_O_3_ ALD is seen clearly after the second ALD cycle, whereas the Al LMM signal was observed from the first ALD cycle as shown in spectra (c)–(g).

**Figure 2:**
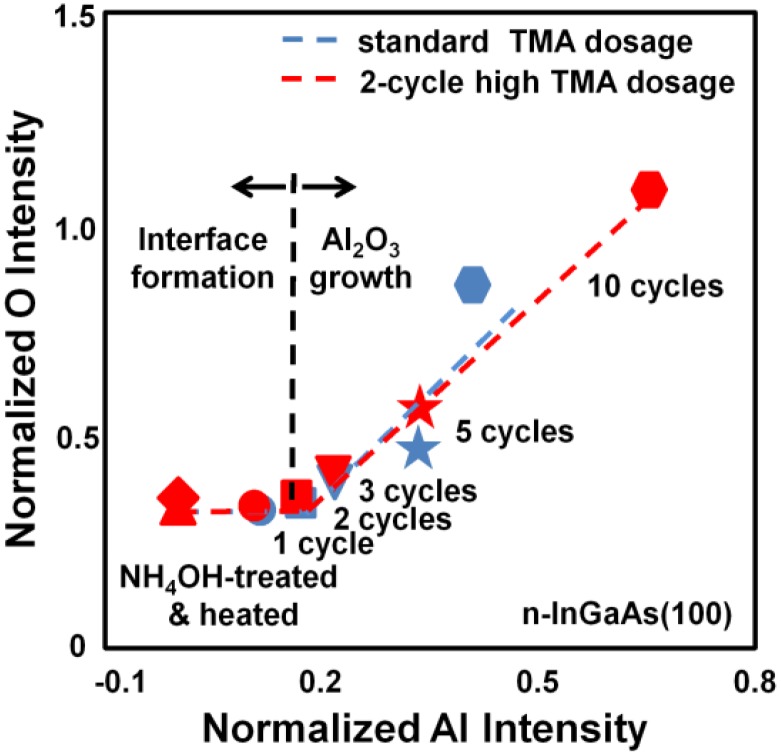
*In situ* AES analysis of the InGaAs(100) surface at energies between the Al LMM and O KLL intensities, normalized to the In MNN intensity, after NH_4_OH treatment, heating at 250 °C, and integrated ALD cycles of 1, 2, 3, 5, and 10.

Figure 2 shows the relationship between the normalized Al LMM and O KLL Auger intensities for a standard TMA dosage and the 2-cycle high TMA dosage conditions. The Auger intensities were quantified by the peak-to-peak intensities of the normalized Al LMM and O KLL signals. The Al signal increased in proportion to the cycle number, whereas the O signal was unchanged in the first and second cycles of both the standard TMA dosage and 2-cycle high TMA dosage conditions. These correlations indicate that Al_2_O_3_ ALD on InGaAs consists of two stages: (1) interface formation (1 to 2 cycles), where the TMA reacts with the InGaAs surface oxide to form the interface and oxygen uptake from the H_2_O vapor does not take place, and (2) bulk growth, in which both the Al and O amounts increase in proportion to the ALD cycle number. These results also show that the Al LMM and O KLL intensities of the 2-cycle high TMA dosage condition are higher than that of the standard dosage conditions after the interface-formation stage as indicated by higher growth rate of Al_2_O_3_ at the beginning of bulk growth. It should be noted that the higher growth rate by high TMA dosage was observed only at the beginning of bulk growth. The growth rate of thick films (~10 nm) was 0.10 nm/cycle, regardless of the TMA dosage.

**Figure 3:**
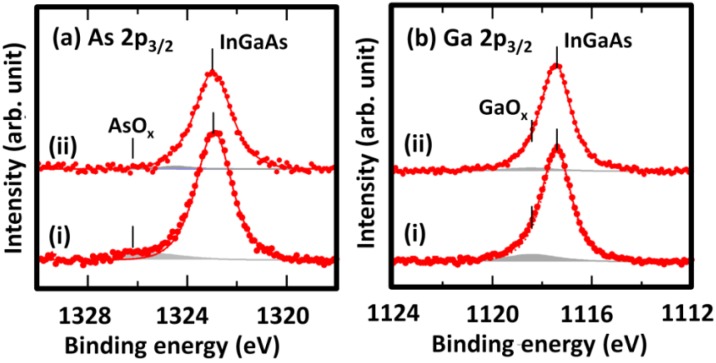

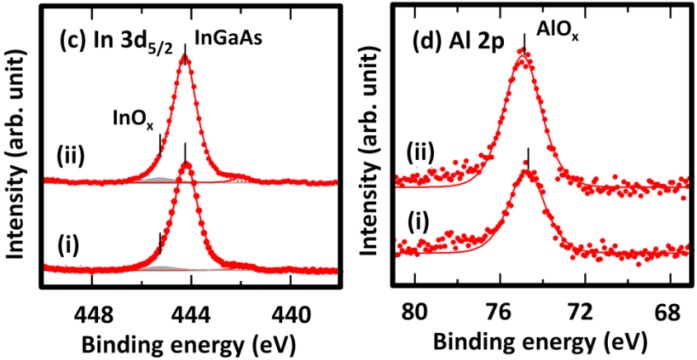
(**a**) As 2p, (**b**) Ga 2p, (**c**) In 3d, and (**d**) Al 2p XPS spectra of Al_2_O_3_ (~1 nm)/InGaAs after ALD with (i) a constant TMA dosage and (ii) a 2-cycle high TMA dosage.

In order to characterize the interfaces formed by the standard TMA dosage and 2-cycle high TMA dosage conditions, InGaAs surfaces with an Al_2_O_3_ cap layer grown by 10-cycle ALD (approximately 1 nm) were analyzed by XPS. Figure 3 shows the data of the As 2p_3/2_, Ga 2p_3/2_, In 3d_5/2_, and Al 2p signals. Spectra (i) and (ii) in each plot label the signal for the standard TMA and 2-cycle high TMA dosages, respectively. The amount of AsO_x_ and GaO_x_ were smaller for the high TMA dosage than the standard dosage. Additionally, the InO_x_ component was too small for us to examine the dosage effects. These XPS results indicate that surface oxide reduction can be promoted by a high TMA dosage during the interface-formation stage. The AlO_x_ intensity of the 2-cycle high TMA dosage conditions is higher than that of the standard dosage and indicated a thicker Al_2_O_3_ film, which corresponds to the higher growth rate as observed in Figure 2. The peak at ~442 eV in the In 3d XPS spectra was reproducibly observed in our XPS measurements of the InGaAs samples. Although this component with a lower binding energy would imply presence of metallic In such as In droplet or surface-segregated In, characterization by AFM and TEM did not show any evidence of such In structures.

In Figure 3, Ga/As intensity ratios for (i) and (ii) were 0.89 and 1.14, respectively. The In/Ga ratios were 0.84 and 1.35 for (i) and (ii), respectively. These data indicate that the high-dosage ALD lead to less As atoms at the MIS interface. As mentioned in introduction, it was reported that the As^5+ ^ oxidation state was detected on the top surface of the Al_2_O_3_ layer. We speculate that such segregation of As oxide to the ALD growth surface was enhanced by the high dosage of TMA.

### 3.2. MIS Properties

**Figure 4:**
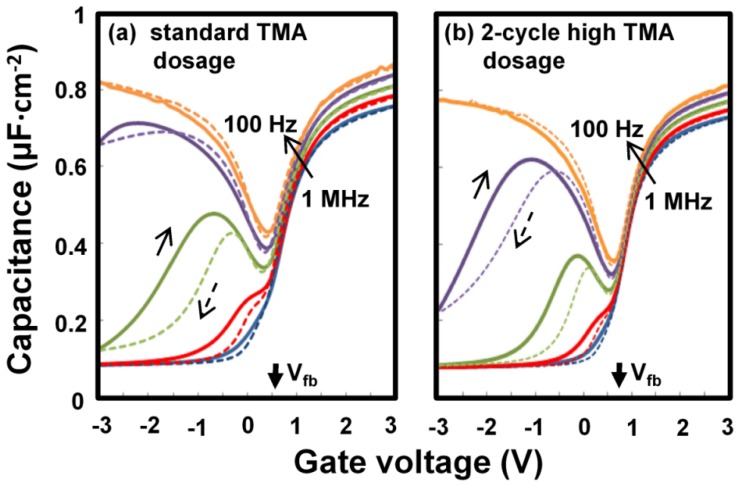
C-V characteristics of Au/Al_2_O_3_ (6 nm)/n-InGaAs(100) capacitors with (**a**) a constant TMA dosage for all 60 cycles, and (**b**) a high TMA dosage for the first 2 cycles and the standard dosage for the remainder. The frequencies were 1 MHz, 100 kHz, 10 kHz, 1 kHz, and 100 Hz as indicated in the figure.

Figure 4 compares the C-V characteristics of the Al_2_O_3_/InGaAs interfaces prepared with (a) the standard TMA dosage for all 60 cycles, and (b) the high dosage for the first two cycles and the standard dosage for the remainder. The C-V curves show that the high TMA dosage caused a positive flatband voltage V_fb_ shift and a reduction of the frequency dispersion around V_fb_.

**Figure 5:**
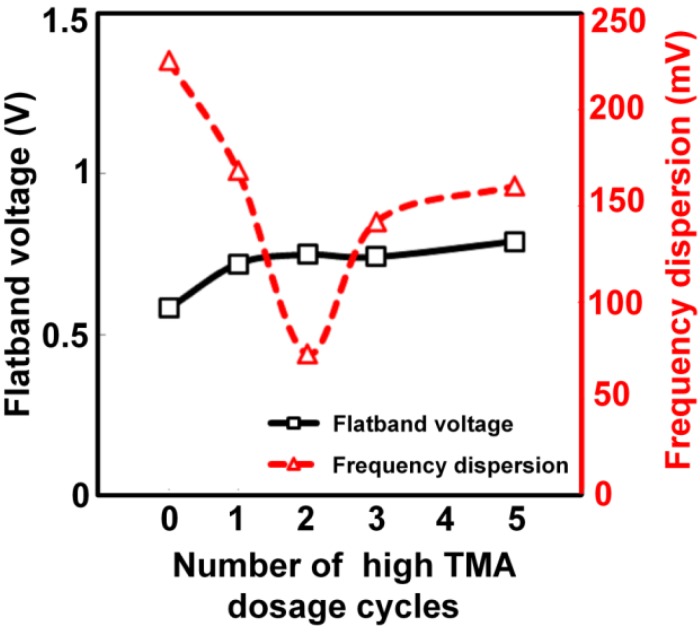
The flatband voltage V_fb_ and frequency dispersion as a function of the number of high-dosage cycles.

Figure 5 shows frequency dispersion and V_fb_ as a function of the number of high-dosage cycles. The frequency dispersion was quantified by measuring the voltage shift at half the maximum capacitance as the frequency was changed from 1 MHz to 100 Hz. Reduction of the frequency dispersion was most remarkable when the high TMA dosage was employed in the first and second cycles, i.e., the interface-formation stage. The minimum frequency dispersion was ~70 mV for a high TMA dosage for the first two cycles, whereas it was as large as 225 mV when the standard dosage was used for all cycles. This smaller frequency dispersion indicates a smaller trap density D_it_. The high-low frequency capacitance method was employed to estimate the D_it_ profile; the minimum D_it_ was 3.1 × 10^12^ cm^−2^eV^−1^ for the standard dosage, and this was reduced to 1.2 × 10^12^ cm^−2^eV^−1^ by using a high TMA dosage for the first two cycles.

**Figure 6:**
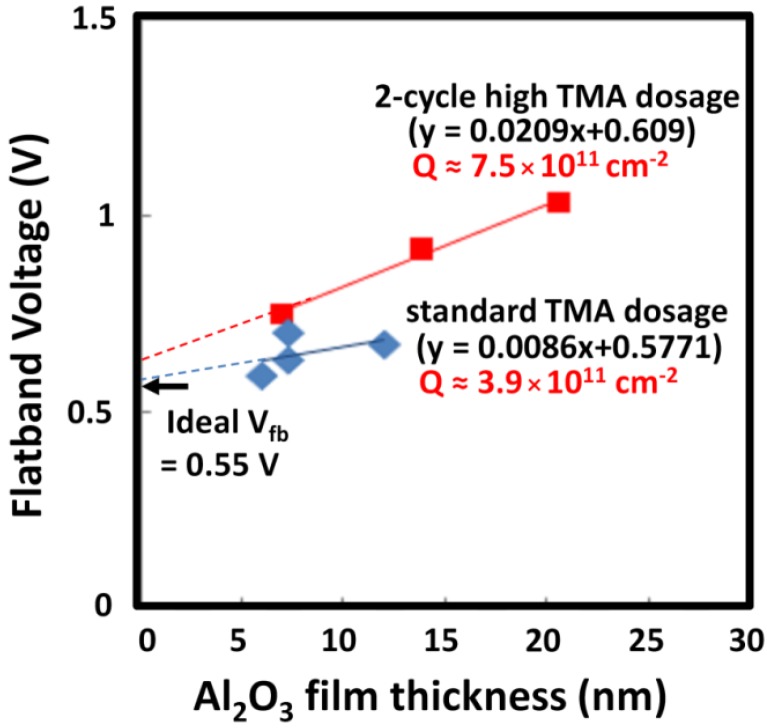
Dependence of the flatband voltage V_fb_ on Al_2_O_3_ thickness. V_fb_ was estimated from the 1 MHz C-V curves of the Au/Al_2_O_3_/n-InGaAs(100) capacitors. The gradient indicates the fixed charge density.

Although the frequency dispersion improved by using the high TMA dosage, the V_fb_ shifted in the positive direction by ~0.2 V. The cause for this shift was examined by measuring the MIS capacitor structures with various Al_2_O_3_ thicknesses. Figure 6 shows the dependence of V_fb_ on the Al_2_O_3_ thickness for both the standard and high TMA dosage conditions. The V_fb_ of the high TMA dosage conditions increased proportionally with the Al_2_O_3_ thickness; however, the dependence was much weaker for the standard dosage. These results indicate that the number of fixed negative charges at the Al_2_O_3_/InGaAs interface is increased by the high TMA dosage during the initial growth. From the gradient of the regression lines, the effective density of the fixed negative charges is estimated to be approximately 3.9 × 10^11^ and 7.5 × 10^11^ cm^−2^ for the standard and high TMA dosage conditions, respectively.

**Figure 7:**
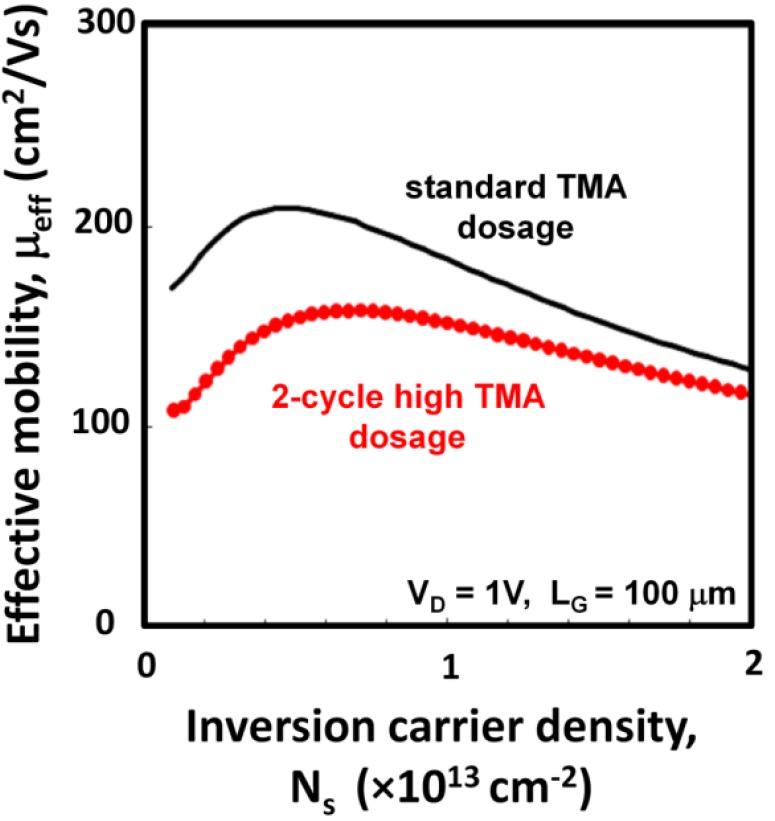
Effective mobilities µ_eff_ of the constant TMA dosage and the 2-cycle high TMA dosage devices. Split C-V data at 1 kHz of the long-channel devices (L_g_/W_g_ = 100/100 µm) were used to estimate the inversion carrier density N_s_.

Figure 7 shows the effective mobility µ_eff_ as a function of the inversion carrier density N_s_. Here, µ_eff_ is defined by the following equation.

µ_eff_ = I_d_ × L_g_/(V_d_ × W_g_ × q × N_s_) (1)

where I_d_ is drain current, L_g_ is channel length, V_d_ is drain voltage, W_g_ is channel width, q is elementary charge and N_s_ was estimated by the split C-V method at 1 kHz from channel dimensions of L_g_/W_g_ = 100/100 µm. While this is a common procedure to evaluate µ_eff_ of MISFETs, estimation of N_s_ may include some error due to the contribution of the trapping states to the C-V measurements. In particular, we recently reported for the InGaAs MISFETs that the trapping states inside the conduction band of InGaAs indeed contribute to the capacitance response, causing an overestimation of N_s_ [20]. Because of this situation, µ_eff_ reported in this paper are indeed *effective* or *apparent* values. In Figure 7, the high TMA dosage device has lower µ_eff_ than that of the standard dosage device. The µ_eff_ at N_s_ = 5 × 10^12^ cm^−2^ was 155 cm^2^/Vs, which is about 26% lower than that of the standard TMA device. There are two possible causes for the lower µ_eff_ by the high TMA dosage. First, the high TMA dosage generated a higher density of the fixed negative charges, as was shown in Figure 6. This would cause stronger carrier scattering via the Coulomb interaction. Second, the MIS interface formed by high TMA dosage could have more trapping states in the bandgap and/or inside the conduction band, and the actual carrier density that contributed to I_d_ was reduced.

## 4. Conclusions

Combining all of the results described above, it can be concluded that the TMA dosage in the interface-formation process is the key to adjusting the ALD-Al_2_O_3_/InGaAs MIS interface properties. The AES results revealed that the initial growth of Al_2_O_3_ consists of two stages: interface formation and bulk growth. This initial 2-cycle Al_2_O_3_ ALD takes place by the reaction between TMA and the InGaAs surface oxides rather than the normal ALD reaction between TMA and H_2_O. XPS results showed that surface oxide reduction can be promoted by a high TMA dosage during the interface-formation stage, and the C-V characteristics indicated that the high TMA dosage is effective in improving the frequency dispersion as it causes a positive shift in V_fb_. Reduction of the frequency dispersion was most remarkable when the high TMA dosage was employed during the first and second cycles. The positive shift of V_fb_ originates from the fixed negative charges at the Al_2_O_3_/InGaAs interface. The device prepared with the high TMA dosage showed a degraded effective carrier mobility as compared with the standard TMA dosage device, and this lower MISFET performance originated from the strong carrier scattering, due to the fixed negative charges at the Al_2_O_3_/InGaAs interface.
